# Nanoparticles based on essential metals and their phytotoxicity

**DOI:** 10.1186/s12951-017-0268-3

**Published:** 2017-04-26

**Authors:** Branislav Ruttkay-Nedecky, Olga Krystofova, Lukas Nejdl, Vojtech Adam

**Affiliations:** 1 Department of Chemistry and Biochemistry, Mendel University in Brno, Zemedelska 1, 613 00 Brno, Czech Republic; 2 Central European Institute of Technology, Brno University of Technology, Technicka 3058/10, 616 00 Brno, Czech Republic

**Keywords:** Agriculture, Fertilizers, Nanomaterials, Essential metal nanoparticles, Nanoparticles uptake, Phytotoxicity

## Abstract

Nanomaterials in agriculture are becoming popular due to the impressive advantages of these particles. However, their bioavailability and toxicity are key features for their massive employment. Herein, we comprehensively summarize the latest findings on the phytotoxicity of nanomaterial products based on essential metals used in plant protection. The metal nanoparticles (NPs) synthesized from essential metals belong to the most commonly manufactured types of nanomaterials since they have unique physical and chemical properties and are used in agricultural and biotechnological applications, which are discussed. The paper discusses the interactions of nanomaterials and vascular plants, which are the subject of intensive research because plants closely interact with soil, water, and atmosphere; they are also part of the food chain. Regarding the accumulation of NPs in the plant body, their quantification and localization is still very unclear and further research in this area is necessary.

## Background

The main issues, of which agriculture worldwide have been facing to, are loss of fertile land due to pollution, desertification and climate changes. Due to unique and outstanding properties of nanomaterials it is not surprising that an effort to improve the agrarian sector using nanotechnology and nanomaterials has been developing [1–11]. Particularly, the use of various types of nanomaterials made of metal oxides, ceramics, silicates, magnetic materials, semiconductor quantum dots (QDs), lipids, polymers, dendrimers, and emulsions [12–15] aims to reduce the applied amount of plant protection products (PPP), to minimize the loss of nutrients during fertilization, and increase revenues through optimized nutrient management in agriculture [3, 4, 16–18].

**Fig. 1:**
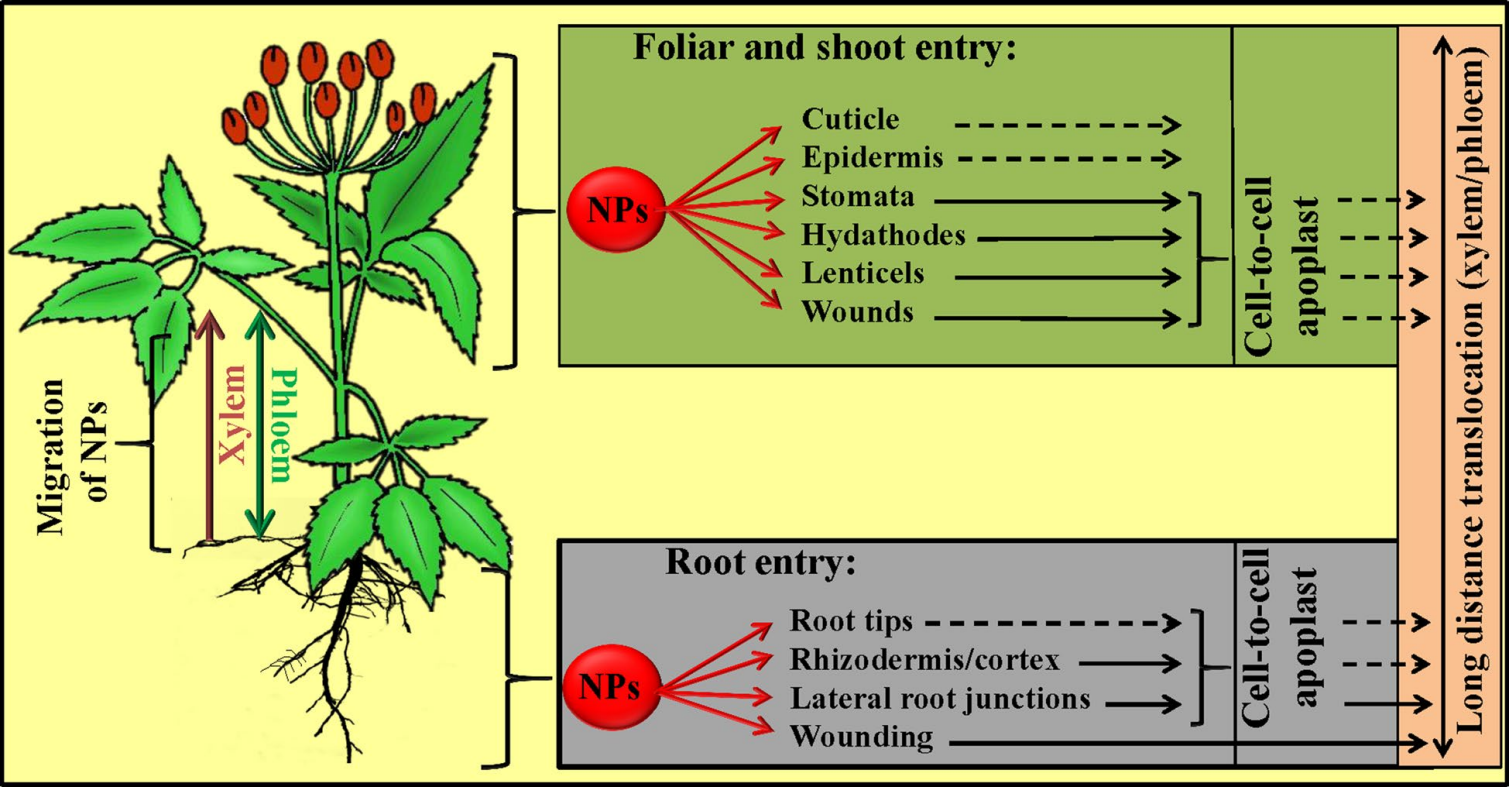
Pathways by which nanoparticles (NPs) are absorbed in plants (Adapted and modified from Dietz et al. [110] and Wang et al. [111])

Greater utilization of nanoparticles (NPs) in agriculture depends on several factors including well known effects, monitored fate as well as their potential toxicity and levels of overdosing. NPs may interact with their environment and plants are a fundamental part of all ecosystems. It can therefore be assumed that NPs will interact with plants and these interactions, such as income and their accumulation in plant biomass, will affect their fate and transport in the environment. NPs may also adhere to the roots of the plants and cause physical or chemical toxicity to plants. Interaction with microorganisms in the soil cannot be excluded because they can positively interact with plants [19–22]. Based on these fact it is clear that there is an ability of nanomaterials to penetrate live plant tissues, but it has ramifications for their accumulation in the food chain and for their utility as smart delivery systems in living plants. Our ability to evaluate these impacts requires an understanding of how NPs are transported within a plant. It is important to understand whether intact NPs can be taken up by plants and transported to other plant tissues. In this area, it was found that NPs can enter plant tissues through either the root tissues or the aboveground organs and tissues (e.g., cuticles, trichomes, stomata, stigma, and hydathodes), as well as through wounds and root junctions (Fig. 1). Only several studies have reported ‘direct’ uptake, translocation, and localization of NPs in plants using various insoluble NPs including mesoporous silica NPs [23], silica NPs (SNPs) [24], carbon nanotubes [25], fullerenes (C70) [26], QDs [27], Au-NPs [28], titanium dioxide NPs (TiO_2_ NPs) [29, 30], iron (II, III) oxide (Fe_3_O_4_) NPs [31, 32], and virus-based NPs [33].

The toxicity of NPs in plants has been discussed several times [8, 34–37]. The conclusions showed that not all plants treated with nanoparticles exhibit toxic effects; substantially more studies showed positive or no consequential effects on plants. Nanotoxicity mechanisms remain unknown, however, it can be assumed to be closely related to the chemical composition, chemical structure, size, and surface area of nanoparticles. The presence of nanoparticles on the root surface can change the surface chemistry of the roots and consequently affect the uptake of nutrients into the plant root [21, 22], thus, these have to be taken into consideration too. Generally, the toxicity of nanoparticles is attributable to two different steps: (1) chemical toxicity based on chemical composition, for example the release of (toxic) ions, and (2) stress stimuli caused by surface, size, or shape of the particles.

In the review, first we describe the basic methods for phytotoxicity testing, then we provide an overview of the most common techniques for detecting and imaging nanoparticles in plants, and then we will focus on the benefits of using essential metal nanoparticles (Zn, Cu, Fe, Mn, and their oxides) in agriculture and current knowledge on their potential phytotoxicity.

## Methods for testing the phytotoxicity of metal nanoparticles

### Phytotoxicity tests

There are no specific test guidelines for nanotoxicity so EPA48 or OECD49 directives from the US for chemical testing are currently used [38]. Phytotoxicity tests generally use plants recommended by these guidelines. These are mostly species of crops, and include both monocotyledonae and dicotyledonae [38]. Species that are recommended most are bean (*Phaseolus vulgaris*), cabbage (*Brassica oleracea*), carrot (*Daucus carota* subsp. *sativus*), cucumber (*Cucumis sativus*), lettuce (*Lactuca sativa*), maize (*Zea mays* subsp. *mays*), oat (*Avena sativa*), onion (*Allium cepa*), radish (*Raphanus sativus*), rice (*Oryza sativa*), ryegrass (*Lolium perenne* L.), soybean (*Glycine max*), tomato (*Solanum lycopersicum*), and wheat (*Triticum aestivum*). Recently, research model species such as the well characterized thale cress (*Arabidopsis thaliana*) were also included [39].

Phytotoxicity tests are carried out in two stages of plant development: (1) during germination, when the germination percentage is measured, where the seeds must be exposed to the test solution for the duration of germination (preferably at least 4 days) [38], and (2) during seedling growth, in which root/shoot elongation and dry weight are frequently used variables to assess the effects of plant exposure to harmful substances [40]. The aforementioned protocols have been applying for testing the effects of nanoparticles in water, wastewater, sediment, and slurry.

**Fig. 2:**
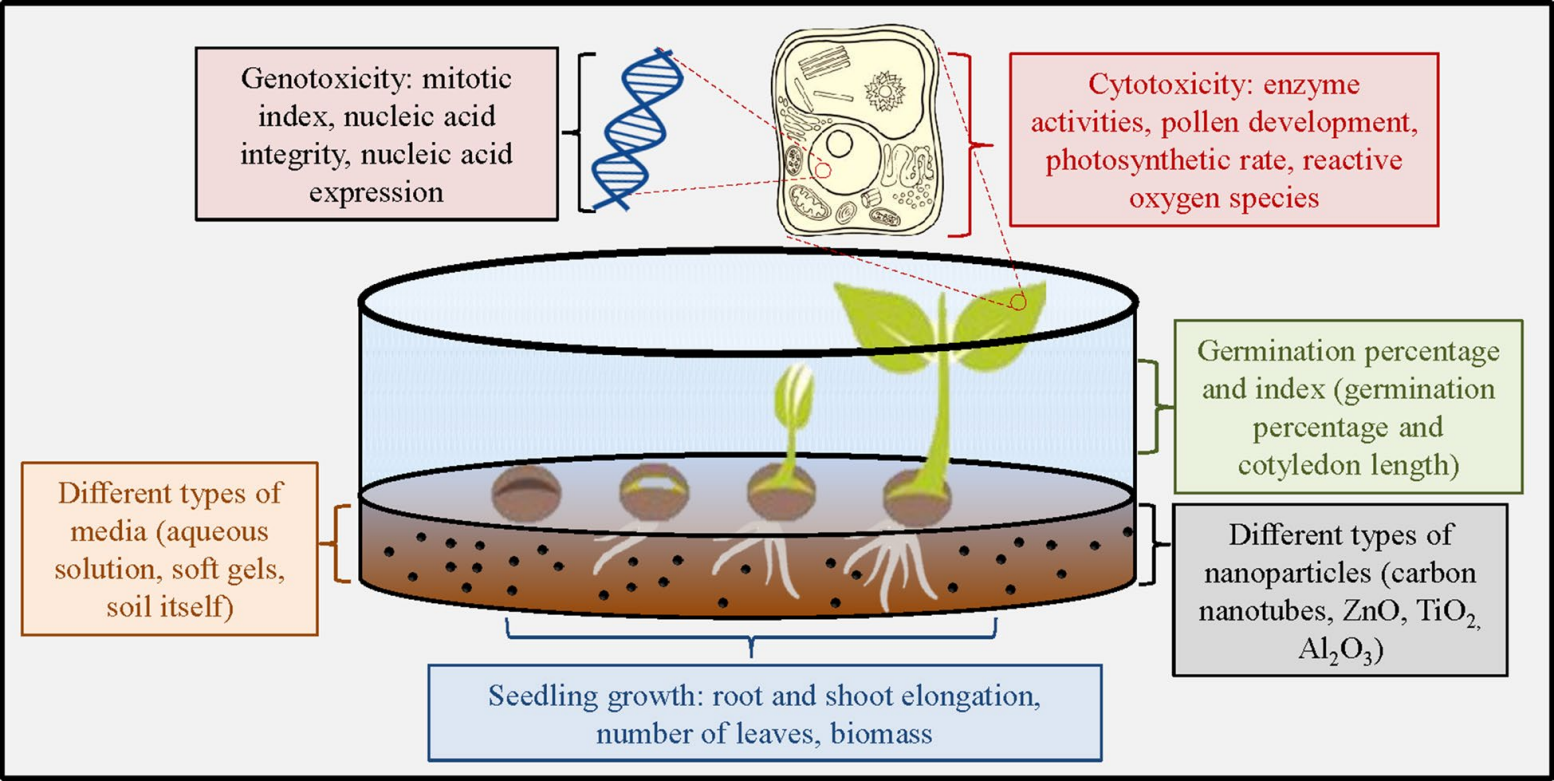
Important considerations when designing phytotoxicity studies and endpoints in phytotoxicity studies (Adapted and modified from Miralles et al. [39])

For phytotoxicity testing different media for the growth of plants are used. The simplest medium is water (Fig. 2). Other applications include soft gels or agars, which better represent the soil, and finally soil itself is also often used [39]. Nanoparticles tend to adsorb to soil matrix and aggregate in the natural environment which reduces their mobility and bioavailability (Fig. 2). Also, the study of the interactions of nanoparticles with plants in alternative substrates does not take into account the potential interaction of nanoparticles with soil and the associated water phase [41]. For example, nanoparticles in soil can influence the growth of soil bacteria, which may then indirectly affect the plant growth [42].

Rico et al. [36] and Peralta-Videa et al. [43] are the pioneers of studies dealing with the effects of nanoparticles on vascular plants. The most common monitored parameters include the germination rate and root/stem growth rate. Recently, the number of leaves [44] and chlorophyll content [45, 46] of exposed plants were included as new monitored parameters for phytotoxicity tests. In addition, the cytotoxicity and genotoxicity of nanoparticles are assessed [39, 47], which is also indicated in Fig. 2.

## Nanomaterials based on essential metals and their use in agriculture

Essential metal nanoparticles are chosen because they are essential metals for plants, and are nontoxic in wide concentration range. This group of metals involves nanoparticles based on Zn, Cu, Fe, Mn, and their oxides. From these, zinc oxide (ZnO) and copper oxide (CuO) nanoparticles (NPs) are used in numerous commercial applications including antimicrobial formulations. Recent studies suggest the use of these NPs as fungicides in agriculture and in the food industry, whereas treatments with ZnO or CuO NPs inhibit the growth of fungal plant pathogens such as *Botrytis cinerea*, *Penicillium expansum, Fusarium graminearum,* and *Phytophthora infestans* [48–50]. Consequently, although NPs may be formulated for use in agriculture as crop protectants, their impact on nontarget soil microbes is not fully known. Both CuO and ZnO NPs cause bacterial cell death at doses that vary with the microorganism [42, 51, 52].

Nanoscale zerovalent iron (Fe^0^) and bimetallic Ni^0^–Fe^0^ nanoparticles have emerged as effective redox media for the detoxification of organic and inorganic pollutants in aqueous solutions. These nanomaterials (10–100 nm) have larger surface areas and reactivity than bulk Fe^0^ particles [53–55]. Mn deficiency has been widely reported all over the world, especially in soils with higher pH (>6.0) or in calcareous, sandy, peat, or muck soils [56]. Therefore, Mn fertilization is very important to improve agronomic production [56]. Manganese nanoparticles (MnNPs) have been also proposed as a suitable alternative to commercially used manganese salts MnSO_4_ (MS) for nanobiotechnology based crop management studies [57].

### Nanomaterials based on Zn

Zinc nanoparticles (NPs) are spherical or polished metal particles with a high specific surface area. The applications of zinc nanocrystals include antimicrobial, antibiotic and antifungal agents, which are a part of painting buildings, dressing materials, nanofibers, plastics, and textiles [58]. Moreover, ZnO NPs are widely used in personal care products such as sunscreens, cosmetics, textiles, lipsticks, and hair dyes. In industrial products they are used in floor coatings, solar cells, as an antibacterial agent, and with optical and electronic materials [59–61]. These sprays are one of the direct routes of ZnO NPs into the environment. ZnO NPs are also present in agricultural spraying as a protecting material against UV radiation [62], where ZnO contributes together with an organic filter for the protection of photosensitive pesticides, is used directly for crop protection against UV radiation [63]. In addition, ZnO NPs have been also studied as a nutrient to increase the efficiency of plant fertilization, but the larger surface area of nanoparticles do not ensure improved solubility or even higher availability of Zn^2+^ for plants [64]. One may suggest that the solubility of Zn^2+^ in Zn fertilizers plays an important role in the agronomic effectiveness of the fertilizer. On the basis of thermodynamics, ZnO NPs should dissolve faster and to a greater extent than bulk ZnO particles (equivalent spherical diameter >100 nm). These novel solubility features of ZnO NPs might be exploited to improve the efficiency of Zn fertilizers. In this field, coated monoammonium phosphate granules show greater Zn solubility and faster dissolution rates in sand columns compared to coated urea granules, which may be related to pH differences in the solution surrounding the fertilizer granules. The kinetics of Zn dissolution was not affected by the size of the ZnO NPs applied for coating of either fertilizer type, possibly because solubility was controlled by the formation of the same compounds irrespective of the size of the original ZnO NPs used for coating [64]. In another study ZnO NPs were investigated for their use as a Zn supplement. Seeds of several plants (*Z. mays*, *G. max*, *Cajanus cajan* and *Abelmoschus esculentum*) were coated with ZnO NPs. The germination test carried out with coated and uncoated seeds indicated a better germination percentage (93–100%) due to the ZnO coating when compared to uncoated seeds (80%). A pot culture experiment was conducted with coated seeds and this also revealed that the crop growth with ZnO coated seeds was similar to that observed with soluble Zn treatment applied as zinc sulfate heptahydrate [47].

Besides supplementation by Zn, ZnO NPs, synthesized by soil fungi in a concentration 10 mg L^−1^, has been shown to enhance the mobilization of native phosphorus in the mung bean (*Vigna radiata*) rhizosphere. The analyses made by authors showed that they synthesized ZnO NPs with average diameter as 22.4 nm as they claimed to have stable nanoparticles due to in situ corona formation by fungal extracellular protein used in the synthesis procedure. Zn acts as a cofactor for phosphorus-solubilizing enzymes such as phosphatase and phytase, and ZnO NPs increased their activity. The level of resultant phosphorus uptake in *V. radiata* increased by 10.8%. In addition, biosynthesized ZnO NPs also improved plant plienology such as stem height, root volume, and biochemical indicators such as leaf protein and chlorophyll contents [65]. Not only the effect but also the way of application was considered by authors as they choose foliar application on 2-week-old mung bean plants. The concentration of the ZnO suspension was 10 mg L^−1^, where a total of 25 mL of suspension ZnO NPs was sprayed on each plant by an atomizer generating droplets. In spite of the foliar application, the aforementioned positive effects have been evidenced.

Next, the fungicide activity of ZnO NPs against *F. graminearum* was investigated, too. Wheat plants were inoculated with *F. graminearum* and treated with ZnO NPs (100 mM). When the wheat plants reached maturation, the grains were harvested and evaluated for *Fusarium* (number of colonies, CFU g^−1^). ZnO NPs showed a reduction in number of CFU of *F. graminearum* when compared to the control [66].

In another work, ZnO NPs were shown to have interactive effects on *Pseudomonas chlororaphis* O6 (PcO6) to inhibit the plant pathogen *F. graminearum.* ZnO NPs were commercial ones with diameter less than 100 nm. Growth of *F. graminearum* was significantly (p = 0.05) inhibited by the inclusion of ZnO NPs in a mung bean both in mung bean agar and in sand tested in the presence and also in no presence of PcO6. The treatment itself lasted for 7 days. The ZnO NPs were significantly more inhibitory to fungal growth than micro-sized particles of ZnO, although both types of particles released similar levels of soluble Zn, indicating size-dependent toxicity of the particles [49].

Thus, one can say that low concentrations of ZnO NPs are beneficial to plants. Positive effects of ZnO NPs are manifested in promoting germination, stem and root growth, increase in phosphorus mobilizing enzymes, phosphorus uptake, and antifungal properties. The observed positive effects of ZnO NPs on plants are summarized in Table 1.

**Table 1:** The observed positive effects of ZnO NPs on plants

Plant	Particle size (nm)	Particle concentration	Comment	Observed effect	References
*Vigna radiata*	22.4 ± 1.8	10 mg L^−1^	NPs were synthesized by soil fungi	Increase in stem height and root length. Increase in phosphorus mobilizing enzymes and phosphorus uptake by 10.8%	[65]
*Zea mays, Glycine max, Cajanas cajan, Abelmoschus esculentus*	<100	25 or 50 mg Zn g^−1^ seed	Seeds were coated with ZnO NPs	Improved germination	[47]
				Coated seeds 93–100%	
				Uncoated seeds 80%	
*Triticum aestivum*	30	100 mM Zn	Wheat plants were inoculated with *Fusarium graminearum*	Reduction in number of CFU of *F. graminearum*	[66]

### Nanomaterials based on copper

Cu/CuO NPs are used in optoelectronics, catalysis, solar cells, as semiconductors, as they are also used as pigments, and fungicides [50, 67, 68]. Copper as fungicide is especially used in vineyards and in organic farming [62]. The ability of copper ions to prevent spore germination of fungi has been known for a long time, but to achieve this effect it is necessary to apply a large amount of copper (500–1500 g ha^−1^). Of note, it is certainly worth a patent of the BASF company [69]. Subject to the patent is the nanoparticulate amorphous Cu^2+^ salt, which forms by a reaction with polymer CuNPs within the size from 1 to 200 nm. Compared with commonly used non-nano product containing cupric hydroxide (Cuprozin, Spiess Urania Chemicals), the same dose of copper in the form of nanoparticles improves efficiency by 8% against a phytopathogenic fungus on vines [62]. This is an example of how the nanoparticle form can reduce the amount of Cu discharged into the environment. Recently, one study demonstrated that CuNPs absorbed in chitosan hydrogel had positive effects on tomato growth and quality [70]. During this process, the activity of some enzymes can increase such as catalase, or decrease in the case of ascorbate peroxidase [71]. It is believed that the stimulatory effects of CuNPs are related to the induction of antioxidant activity [72]. The positive effect of CuNPs on *S. lycopersicum* is shown in Table 2 in the same way as in the case of Zn NPS.

**Table 2:** The observed positive effects of CuNPs on *Solanum lycopersicum*

Plant	Particle size (nm)	Particle concentration	Comment	Observed effect	Reference
*Solanum lycopersicum*	<100	15, 30, 60, 150 mg L^−1^	CuNPs were adsorbed on chitosan hydrogels	Application of chitosan hydrogels with CuNPs was favorable to tomato growth and quality	[70]

### Nanomaterials based on iron

Iron nanoparticles (INPs) represent a new generation of environmental remediation technologies that could provide cost-effective solutions to some of the most challenging environmental issues. Because of large surface areas and high surface reactivity [73], INPs have found their main application in remediation [71]. This method is relatively cheap and uses both free (soil application, where INPs can penetrate ground water) and into matrix fixed nanoparticles (cleaning water or air) [54]. In the greatest extent INPs are used to decompose substances such as chlorinated hydrocarbons (e.g. trichloroethylene), organochlorine pesticides, and polychlorinated biphenyls [54]. Besides decomposing, INPs can be further applied to bind, for example, to a significant pollutant, arsenic ions [74]. Materials composed of nanoscaled iron particles exhibit high absorbency and a second advantage is their response to external magnetic fields by which they can be, even with bound arsenic compounds, removed. The mentioned procedure can also be used for other metals such as mercury or lead [75].

In agriculture, Fe_2_O_3_ NPs may be used instead of Fe fertilizers [76]. Rui et al. evaluated the effectiveness of iron oxide nanoparticles (IONPs; Fe_2_O_3_ NPs) as a fertilizer to replace traditional Fe fertilizers [77]. The effects of the Fe_2_O_3_ NPs and a chelated-Fe fertilizer (ethylenediaminetetraacetic acid-Fe; EDTA-Fe) on the growth and development of peanut (*Arachis hypogaea*), a crop that is very sensitive to Fe deficiency, were studied in a pot experiment. The results showed that Fe_2_O_3_ NPs increased root length, plant height, biomass, and soil plant analysis development (SPAD) values of peanut plants. The Fe_2_O_3_ NPs promoted the growth of peanuts by regulating phytohormone contents and antioxidant enzyme activity. The Fe contents in peanut plants with Fe_2_O_3_ NPs and EDTA-Fe treatments were higher than the control group. The next study was conducted to examine the effect FeNPs (prepared by reduction with a gum kondagogu) on the growth of a mung bean (*V. radiata*). The radical length and biomass was increased in seeds exposed to FeNPs in comparison with the ions [78]. In the following study, the uptake of iron oxide (Fe_2_O_3_) nanoparticles by spinach (*Spinacea oleracea*) via hydroponics was demonstrated and its effects on the growth rate and productivity of the spinach plant were examined. The experimental studies such as plant growth (stem and root length) and biomass analysis revealed a dose and time dependent increase due to the uptake of Fe_2_O_3_ [19]. In the next study, Trujillo-Reyes et al. showed that iron NPs, unlike CuNPs, did not affect the chlorophyll content, plant growth, catalase (CAT), and ascorbate peroxidase (APX) activities of lettuce (*L. sativa*) [71].

In another work, INPs after foliar application had significant effect on yield, leaf Fe content, stem Mg content, plasma membrane stability, and chlorophyll content of *Vigna unguiculata* [79]. In the following study, Alidoust et al. investigated the effect of 6-nm IONPs and citrate-coated IONPs (IONPs-Cit) on photosynthetic characteristics and root elongation during germination of a soybean (*G. max* L.) [20]. Plant physiological performance was assessed after foliar and soil IONPs fertilization. No adverse impacts at any growth stage of the soybeans were observed after the application of IONPs. The Fe_2_O_3_ nanoparticles produced a significant positive effect on root elongation, particularly when compared to the bulk counterpart (IOBKs) suspensions of concentrations greater than 500 mg L^−1^. In the next study of Ghafariyan et al. [80] seed germination of a soybean exposed to superparamagnetic iron oxide nanoparticles (SPIONs) was investigated. It was found that SPIONs, which were entered and translocated in the soybean, increased chlorophyll levels with no trace of toxicity. Furthermore, it was found that physicochemical characteristics of the SPIONs had a crucial role in the enhancement of chlorophyll content in subapical leaves of soybeans. The equivalent ratio of chlorophyll *a* to *b* in all treatments with conventional growth, medium iron chelate, and SPIONs (as iron source) indicated no significant difference on the photosynthesis efficiency. An overview of the positive effects of INPs and iron oxide nanoparticles (IONPs) on plants is shown in Table 3.

**Table 3:** The observed positive effects of iron/iron oxide NPs on plants

Plant	Particle size (nm)	Particle concentration	Comment	Observed effect	References
*Arachis hypogaea*	γ-Fe_2_O_3_, 20 nm	2, 10, 50, 250, 1000 mg kg^−1^ of soil	Fe_2_O_3_ NPs were applied into soil and compared with a chelated-Fe fertilizer	Fe_2_O_3_ NPs increased root length, plant height, biomass, and SPAD values of peanut plants. Fe_2_O_3_ NPs adsorbed onto sandy soil and improved the availability of Fe to the plants. Fe_2_O_3_ NPs can replace traditional Fe fertilizers in the cultivation of peanut plants	[77]
*Vigna radiata*	FeNPs 2–6 nm +0.2% gum, +0.4% gum	1 mM Fe^2+^ions	Natural biopolymer gum kondagogu as reducing and capping agent was used	The radical length and biomass was increased in seeds exposed to Fe NPs in comparison to Fe^2+^ ions. The α-amylase activity was increased in the seeds exposed to Fe NPs	[78]
*Spinacea oleracea*	α-Fe_2_O_3_ 50 nm	100, 150, 200 mg kg^−1^ of soil	Experiments were performed in a solid hydroponic medium consisting of sawdust and coco peat and adequate amounts of water	Positive effects on spinach plant due to uptake of Fe_2_O_3_ nanoparticles such as increase in stem and root lengths, biomass production and magnetic properties were observed	[19]
*Lactuca sativa*	Core–shell NPs Fe/Fe_3_O_4_ 13/9 nm	10, 20 mg L^−1^	15-days treatment of hydroponically grown lettuce	The nano-Fe/Fe_3_O_4_ at 10 and 20 mg L^−1^ and FeSO_4_·7H_2_O at 10 mg L^−1^ did not affect lettuce growth and chlorophyll content	[71]
*Vigna unguiculata*	<100 nm	25, 500 mg L^−1^	The elements were applied 56 and 72 days after sowing over the leaves, and data was collected after day 85	Iron had significant effect on yield, leaf Fe content, stem Mg content, plasma membrane stability, and chlorophyll content, probably as a result of more efficient photosynthesis	[79]
*Glycine max*	γ-Fe_2_O_3_ (IONPS) and citrate coated IONPs 6 nm	500, 1000 mg L^−1^	Plant physiological performance was assessed after foliar and soil IONPs fertilization	IONPs produced a significant positive effect on root elongation. IONPs-Cit significantly enhanced photosynthetic parameters when sprayed foliarly. More pronounced positive effects of IONPs via foliar application than by soil treatment was observed	[20]
*Glycine max*	Superparamagnetic iron oxide NPs (SPIONs) 8–12 nm	200, 400, 1000 and 2000 mg L^−1^	Seed germination of soybean exposed to SPIONs was investigated	SPIONs, which were entered and translocated in the soybean, increased chlorophyll levels, with no trace of toxicity	[80]

### Nanomaterials based on manganese

Manganese (Mn) is an essential micronutrient for growth regulation and the development of plants [81]. It plays a pivotal role in oxygenic photosynthesis both directly and indirectly. The major drawbacks associated with Mn deficiency are plant nutritional disorders [81]. To circumvent this nutritional disorder of plants, nanoparticle mediated crop management has of late found potential applications [57].

In a study by Pradhan et al. the effect of manganese nanoparticles (MnNPs) on nitrogen uptake in mung bean plants (*V. radiata*) was investigated [82]. The objective of this study was to determine the response of manganese nanoparticles (MnNP) in nitrate uptake, assimilation, and metabolism compared with the commercially used manganese salt, manganese sulfate (MS). MnNPs were modulated to affect the assimilatory process by enhancing the net flux of nitrogen assimilation through NR-NiR and GS-GOGAT pathways. This study was associated with toxicological investigation on in vitro and in vivo systems to promote MnNPs as a nanofertilizer and can be used as an alternative to MS.

In another study from the same research group [57] MnNP-treated chloroplasts showed greater photophosphorylation, oxygen evolution with respect to control, and MnSO_4_-treated chloroplasts as determined by biophysical and biochemical techniques. Positive effects on root and shoot elongation was observed. MnNP-treated plants did not trigger oxidative stress.

In the next study, Liu et al. [83] investigated the effects of laboratory-prepared MnOx NPs on the germination of lettuce (*L. sativa*) seeds in a water medium. MnOx NPs only slightly reduced the germination percentage from 84% (control) to 63% even at a high concentration of 50 mg L^−1^ and was not significantly different from that of the control. Furthermore, MnOx NPs specifically improved the growth of lettuce seedlings by enhancing root elongation. For example, the 5-day root length of the seedlings increased by 68%. Similarly, 10- and 5-mg L^−1^ NPs also significantly increased the elongations by 41.6 and 53.9%, respectively. An overview of the positive effects of MnNPs and manganese oxide nanoparticles (MnOx NPs) in plants is shown in Table 4.

**Table 4:** The observed positive effects of MnNPs on plants

Plant	Particle size (nm)	Particle concentration	Comment	Observed effect	References
*Vigna radiata*	MnNPs	50, 100, 500, 1000 mg L^−1^	Leaf and root enzyme extract was analyzed for use as nanofertilizer	Nitrogen uptake, its assimilation, and metabolism was increased after MnNPs soil application	[82]
*Vigna radiata*	MnNPs	50, 100, 500, 1000 mg L^−1^	Leaf and root enzyme extract was analyzed. Chloroplasts from leaves were isolated and analyzed for their level of photophosphorylation and oxygen evolution	MnNP-treated chloroplasts showed greater photophosphorylation, oxygen evolution with respect to control and MnSO4-treated chloroplasts. Positive effects on root and shoot elongation was observed	[57]
*Lactuca sativa*	MnOx NPs 5–15 nm	0.25, 0.5, 5, 10, mg L^−1^	Overall, the data suggests that MnOx NPs can be used as an Mn fertilizer (better than their soluble or bulk solid counterparts) for crop growth improvement	MnOx NPs specifically improved the growth of lettuce seedlings by enhancing root elongation	[83]

## Phytotoxicity of ZnO, Cu (CuO), and iron oxide nanoparticles

A good understanding of the mechanisms of the nanoparticle phytotoxicity is important for the targeted application of nanoparticles [84]. Essential metal NPs can cause phytotoxicity via the dissolution and release of higher concentration of essential ions [85, 86] such as Zn^2+^ and Cu^2+^ or the production of excess reactive oxygen species (ROS) through redox cycling and the Fe^2+^-mediated Fenton reaction [87].

### Phytotoxicity of nanoparticles based on ZnO

Ecotoxicity studies on ZnO NPs are most abundant in bacteria and are relatively lacking in other species [88]. These studies suggest relative high acute toxicity of ZnO NPs (in the low mg L^−1^ levels) to environmental species, although this toxicity is highly dependent on test species, physicochemical properties of the material, and test methods. Particle dissolution to ionic zinc and particle-induced generation of ROS represent the primary modes of action for ZnO NPs toxicity across all species tested, and photo-induced toxicity associated with its photocatalytic property may be another important mechanism of toxicity under environmentally relevant UV radiation [85].

ZnO NPs have been shown to induce oxidative stress in soybean (*G. max*) seedlings in a concentration of 500 mg L^−1^. Plant growth, rigidity of roots, and root cell viability were markedly affected by ZnO NPs stress. Oxidation–reduction cascade related genes, such as GDSL motif lipase 5, SKU5 similar 4, galactose oxidase, and quinone reductase were down-regulated in ZnO NPs treatment [89].

In the next study, Mukherjee et al. [90] investigated the impact of different zinc oxide (ZnO) NPs on green pea plants (*Pisum sativum* L.). Pea plants were grown for 65 days in soil amended with commercially available bare ZnO NPs (10 nm), 2 wt% alumina doped Al_2_O_3_/ZnO NPs (15 nm), or 1 wt% aminopropyltriethoxysilane coated KH550/ZnO NPs (20 nm) at 250 and 1000 mg NPs.kg^−1^ soil inside a greenhouse. Although all treated plants showed higher tissue Zn content than controls, those exposed to Al_2_O_3_/ZnO NPs at 1000 mg kg^−1^ had greater Zn concentration in roots and seeds, compared to bulk Zn and the other NPs treatments. In leaves, Al_2_O_3_/ZnO NPs at 250 mg kg^−1^ significantly increased chlorophyll-a and carotenoid concentrations relative to the bulk, ionic, and other NPs treatments. The protein and carbohydrate profiles remained largely unaltered across all treatments with the exception of Al_2_O_3_/ZnO NPs at 1000 mg kg^−1^ where the sucrose concentration of green peas increased significantly, which is likely a biomarker of stress. Most importantly, these findings demonstrate that lattice and surface modification can significantly alter the fate and phytotoxic effects of ZnO NPs in food crops and seed nutritional quality.

In another study, ZnO NPs at concentrations of 2000 mg L^−1^ have been shown to inhibit root elongation (50.45% for maize and 66.75% for rice) of two crop plants [91]. Similarly, Xiang et al. [92] observed that ZnO NPs did not affect germination rates at concentrations of 1–80 mg L^−1^ but significantly inhibited the root and shoot elongation of Chinese cabbage seedlings, with the roots being more sensitive. Both the production of free hydroxyl groups and the Zn bioaccumulation in roots or shoots resulted in toxicity of ZnO NPs to Chinese cabbage seedlings. In another work, the impact of ZnO NPs on rhizobium-legume symbiosis was studied with the garden pea (*P. sativum*) and its compatible bacterial partner *Rhizobium leguminosarum*. Exposure of peas to ZnO NPs (500–1000 mg L^−1^) had no impact on germination, but significantly affected root length. Chronic exposure of the plant to ZnO NPs impacted its development by decreasing the number of the first- and the second-order lateral roots, stem length, leaf surface area, and transpiration. Exposure of *R. leguminosarum by. viciae 3841* to ZnO NPs brought about morphological changes by rendering the microbial cells toward round shapes and damaging the bacterial surface. Furthermore, the presence of ZnO NPs in the rhizosphere affected root nodulation, delayed the onset of nitrogen fixation, and caused early senescence of nodules. The attachment of NPs on the root surface and dissolution of Zn^2+^ are important factors affecting the phytotoxicity of ZnO NPs, hence, the presence of ZnO NPs in the environment is potentially hazardous to the rhizobium-legume symbiosis system [93].

Wang et al. used synchrotron-based techniques to examine the uptake and transformation of Zn in various tissues of cowpea [*V. unguiculata* (L.) Walp.] exposed to ZnO NPs or ZnCl_2_ following growth in either a solution or soil culture. In the solution culture, soluble Zn (ZnCl_2_) was more toxic than the ZnO NPs, although there was a substantial accumulation of ZnO NPs on the root surface. When grown in soil, however, there was no significant difference in plant growth and accumulation or speciation of Zn between soluble Zn and ZnO NPs treatments, indicating that the added ZnO NPs underwent rapid dissolution following their entry into the soil [94].

In next study, the effect of exposure to 100 mg L^−1^ ZnO NPs on gene expression in *A. thaliana* roots was studied using microarrays. The genes induced by ZnO NPs include mainly ontology groups annotated as stress responsive, including both abiotic (oxidative, salt, water deprivation) and biotic (wounding and defense to pathogens) stimuli. The down-regulated genes upon ZnO NPs exposure were involved in cell organization and biogenesis, including translation, nucleosome assembly, and microtubule based process [95].

In another study, soybean plants (*G. max*) were grown through the seed production stage in soil amended with ZnO NPs (0, 50, 100 or 500 mg kg^−1^). Although ZnO NPs slightly stimulated plant growth, most striking was the degree to which Zn bioaccumulated in all tissues and especially in the leaves. Zn that translocated aboveground in the present study may have been substantially dissolved from the ZnO NPs added to the soil. This study shows that two high productions of ZnO NPs are able to change soybean agriculture, and demonstrates the importance of managing waste streams to control such exposures [96]. In the following work, the effects of ZnO NPs on the soil plant interactive system were estimated. The growth of plant seedlings in the presence of different concentrations of ZnO NPs within microcosm soil (M) and natural soil (NS) was compared. Changes in dehydrogenase activity (DHA) and soil bacterial community diversity were estimated based on the microcosm with plants (M + P) and microcosm without plants (M − P) in different concentrations of ZnO NPs treatment. The shoot growth of M + P and NS + P was significantly inhibited by 24 and 31.5% relative to the control at a ZnO NPs concentration of 1000 mg kg^−1^. The DHA levels decreased following increased ZnO NPs concentration. Specifically, these levels were significantly reduced from 100 mg kg^−1^ in M − P and only 1000 mg kg^−1^ in M + P [21].

Dimkpa et al. [67] investigated the impact of commercial ZnO (<100 nm) NPs on wheat (*T. aestivum*) grown in a solid matrix, sand. Solubilization of metals occurred in the sand at similar rates from ZnO NPs as their bulk equivalents. Amendment of the sand with Zn (500 mg kg^−1^) from the ZnO NPs significantly (p = 0.05) reduced root growth, growth reduction was less with the bulk amendment. Bioaccumulation of Zn as Zn-phosphate was detected in the shoots of ZnO NP-challenged plants. Oxidative stress in the ZnO NPs-treated plants was evidenced by increased lipid peroxidation and oxidized glutathione in roots and decreased chlorophyll content in shoots; higher peroxidase and catalase activities were present in roots. These findings correlate with the ZnO NPs causing increased production of ROS.

The next study was carried out to examine the phytotoxicity and oxidative stress by ZnO NPs in cucumber (*Cucumis sativus*). Kim et al. [97] estimated the bioaccumulation of ZnO NPs in plants, reactive oxygen species enzyme [superoxide dismutase (SOD), catalase (CAT), and peroxidase (POD)] activities in plant root tissue, and observed ZnO NPs with transmission electron microscopy. They found that the seedling biomass significantly decreased to 35% of that of control at 1000 mg L^−1^ of ZnO NPs. The median inhibition concentrations of ZnO NPs were 215 mg L^−1^. In transmission electron microscopy, ZnO NPs greatly adhered to the root cell wall and some of the ZnO NPs were observed in the root cells. Another finding indicated that ZnO NPs caused statistically significant increases in SOD, CAT, and POD activities and a significant increase already at 100 mg L^−1^ concentration levels. These results indicate that ZnO NPs altered both phytotoxicity and oxidative stress in plant assays.

In the following study the effects of zinc oxide NPs (ZnO NPs) on the root growth, root apical meristem mitosis, and mitotic aberrations of garlic (*Allium sativum* L.) were investigated. ZnO NPs caused a concentration-dependent inhibition of root length. When treated with 50 mg L^−1^ ZnO NPs for 24 h, the root growth of garlic was completely blocked. The 50% inhibitory concentration (IC50) was estimated to be 15 mg L^−1^. ZnO NPs also induced several kinds of mitotic aberrations, mainly consisting of chromosome stickiness, bridges, breakages, and laggings. The total percentage of abnormal cells increased with the increase of ZnO NPs concentration and the prolonging of treatment time. The investigation provided new information for the possible genotoxic effects of ZnO NPs on plants [98].

In the next study, transport of ZnO NPs in a sandy loam soil and their uptake by corn plants (*Z. mays*) was investigated. Results showed that ZnO NPs exhibit low mobility in a soil column at various ionic strengths. By using an electron microprobe, Zn/ZnO NPs aggregates were visualized associating them with soil clay minerals. The uptake (mg kg^−1^) of Zn by 1-month old corn plants varied from 69 to 409 in roots and from 100 to 350 in shoots, respectively, in soils contaminated with different concentrations of ZnO NPs (from 100 to 800 mg kg^−1^ soil). Confocal microscope images showed that ZnO NPs aggregates penetrated the root epidermis and cortex through the apoplastic pathway, however, the presence of a few NP aggregates in xylem vessels suggests that the aggregates passed the endodermis through the symplastic pathway. Most of the aggregates, however, remained around the endodermis border [99]. An overview of the phytotoxicity of nanoparticles based on ZnO is shown in Table 5.

**Table 5:** Phytotoxicity of nanoparticles based on ZnO

Plant	Type of nanoparticle, particle size (nm)	Particle concentration	Comment	Observed effect	References
*Glycine max*	ZnO NPs <50 nm	500 ppm	Effect of ZnO NPs on soybean seedlings was studied	Decrease in root growth (length and weight), loss of root cell viability, accumulation of superoxide and decrease in leaf weight, down regulation of oxidative cascade related genes	[89]
*Pisum sativum*	Bare ZnO NPs 10 nm, Al_2_O_3_/ZnONPs 15 nm, KH550/ZnO NPs 20 nm	250, 1000 mg L^−1^ of soil	Pea plants were grown for 65 days in soil amended with three types of ZnO NPs	Al_2_O_3_/ZnO NPs at 250 mg kg^−1^ significantly increased chlorophyll-a and carotenoid concentrations. Al_2_O_3_/ZnO NPs at 1000 mg kg^−1^ significantly increased sucrose concentration of green peas	[90]
*Zea mays, Oryza sativa*	ZnO NPs <50 nm	500, 1000, 2000 mg L^−1^	Seed germination was investigated	ZnO NPs inhibited root elongation at 2000 mg L^−1^ (50.45% for maize and 66.75% for rice) of two crop plants	[91]
*Brassica pekinensis*	Spheric ZnO NPs 30 nm, spheric ZnO NPs 50 nm, columnar ZnO NPs 90 nm, hexagon rod-like ZnO NPs 150 nm	1, 5, 10, 20, 40, 80 mg L^−1^	There were no significant differences in observed effects between different NPs	ZnO NPs inhibited the root and shoot elongation of Chinese cabbage seedlings. The highest inhibition of root elongation at 80 mg L^−1^ was observed	[92]
*Pisum sativum*	ZnO NPs <50 nm	250, 500, 750, 1000 mg L^−1^	No impact on germination	ZnO NPs (500–1000 mg L^−1^) significantly inhibited root elongation	[93]
*Vigna unguiculata*	ZnO NPs <100 nm	25 mg L^−1^	More pronounced effects were observed with ZnCl_2_ than with ZnO NPs	Significant decrease in biomass production of roots and leaves observed in solution culture, but not observed in soil culture	[94]
*Arabidopsis thaliana*	ZnO NPs <100 nm	100 mg L^−1^	Effect of ZnO NPs on gene expression in plant roots were studied	Induction of stress responsive genes, down regulation of genes involved in cell organization and biogenesis	[94]
(*Glycine max*)	ZnO NPs 10 nm	50, 100, and 500 mg kg^−1^ of soil	ZnO NPs were added to the soil	Zn bioaccumulated in all tissues and especially in the leaves	[96]
*(Fagopyrum esculentum)*	ZnO NPs <50 nm	10, 100, and 1000 mg kg^−1^ of soil	ZnO NPs were added to the soil and growth of plant seedlings were observed	Inhibition of shoot growth	[21]
*Triticum aestivum*	ZnO NPs <100 nm	500 mg kg^−1^ sand	ZnO NPs were added to the sand	Reduced root growth, increased lipid peroxidation and oxidized glutathione in roots. Bioaccumulation of Zn and decreased chlorophyll content in the shoots	[67]
*Cucumis sativus*	ZnO NPs 50 nm	10, 50, 100, 500, 1000 mg L^−1^	Hydroponic experiments	Decrease in seedling biomass. ZnO NPs adhered to the root cell wall, and some of them were observed in the root cells	[97]
*Allium sativum*	ZnO NPs 3–5 nm	10, 20, 30, 40, 50 mg L^−1^	Hydroponic experiments	Concentration-dependent inhibition of root length, observed mitotic aberrations	[98]
*Zea mays*	ZnO NPs 370–410 nm	20 mg L^−1^	ZnO NPs were added to the sandy loam soil or to the water	ZnO NPs aggregates penetrated the root epidermis and cortex. Some of the NPs aggregates were also present in xylem vessels	[112]

### Phytotoxicity of nanoparticles based on Cu/CuO

CuNPs toxicity mechanisms have been extensively studied in animal/human systems [100, 101]. In plants, toxicity of Cu and Cu ions was thoroughly investigated [102] but not so Cu/CuO NPs phytotoxicity [103]. Zhao et al. [104] investigated the response of cucumber plants in hydroponic culture at early development to two concentrations of CuNPs (10 and 20 mg L^−1^). Results showed that CuNPs interferes with the uptake of a number of micro- and macronutrients such as Na, P, S, Mo, Zn, and Fe. Metabolomics data revealed that CuNPs at both levels triggered significant metabolic changes in cucumber leaves and root exudates. The root exudate metabolic changes revealed an active defense mechanism against CuNPs stress: up-regulation of amino acids to sequester/exclude Cu/CuNPs, down regulation of citric acid to reduce the mobilization of Cu ions, ascorbic acid up-regulation to combat reactive oxygen species, and up-regulation of phenolic compounds to improve antioxidant system. It also observed a decrease in root length, reduction of root biomass, and bioaccumulation of Cu mainly in roots.

The following study was conducted to assess the effects of laboratory-prepared CuNPs in low concentrations (<50 ppm) on the germination of lettuce (*L. sativa*) seeds in a water medium. The data showed that CuO NPs were slightly more toxic than Cu^2+^ ions and a reduction of seed germination and root elongation was observed [83]. In the next study 18-day-old hydroponically grown lettuce (*L. sativa*) seedlings were treated for 15 days with core–shell Cu/CuO NPs at two concentrations (10 and 20 mg L^−1^). The results showed that Cu^2+^ ions or Cu/CuO NPs reduced water content, root length, and dry biomass of the lettuce plants. ICP-OES results showed that Cu/CuO NPs treatments produced significant accumulations of Cu in roots compared to the Cu^2+^ ions. In roots, all Cu treatments increased CAT activity but decreased APX activity. In addition, relative to the control, nano-Cu/CuO altered the nutritional quality of the lettuce, since the treated plants had significantly more Cu, Al, and S, but less Mn, P, Ca, and Mg [71].

In another study Atha et al. reported that copper oxide nanoparticles induced DNA damage in agricultural and grassland plants. Significant accumulation of oxidatively modified, mutagenic DNA lesions (7,8-dihydro-8-oxoguanine; 2,6-diamino-4-hydroxy-5-formamidopyrimidine; 4,6-diamino-5-formamidopyrimidine) and strong plant growth inhibition was observed for radish (*R. sativus*), perennial ryegrass (*L. perenne*), and annual ryegrass (*Lolium rigidum*) under controlled laboratory conditions [105].

The next study investigated the phytotoxicity and accumulation of copper oxide (CuO) NPs to *Elsholtzia splendens* (a Cu-tolerant plant) under hydroponic conditions. The Cu content in the shoots treated with 1000 mg L^−1^ CuO NPs was much higher than those exposed to the comparable 0.5 mg L^−1^ soluble Cu and CuO bulk particles. CuO NPs-like deposits were found in the root cells and leaf cells. Cu K-edge X-ray absorption near-edge structure analysis further revealed that the accumulated Cu species existed predominantly as CuO NPs in the plant tissues. All these results suggested that CuO NPs can be absorbed by the roots and translocated to the shoots in *E. splendens*.

In another study, phytotoxicity of CuO NPs was assessed in two crop plants, maize (*Z. mays*) and rice (*O. sativa*). The results showed that seed germination was not affected by CuO NPs at any of the investigated concentrations. However, at the concentration of 2000 mg L^−1^, the root elongation was significantly inhibited by CuO NPs (95.73% for maize and 97.28% for rice), and the shoot length of maize was reduced by 30.98%. An overview of phytotoxicity of nanoparticles based on Cu/CuO is shown in Table 6.

**Table 6:** Phytotoxicity of nanoparticles based on Cu/CuO

Plant	Type of nanoparticle, particle size (nm)	Particle concentration	Comment	Observed effect	References
*Cucumis sativus*	CuNPs 40 nm	10, 20 mg L^−1^	Analysis of plants and root exudates	Decrease in root length, reduction of root biomass, bioaccumulation mainly in roots, a little in stems	[104]
*Lactuca sativa*	CuO NPs 5–15 nm	0.02, 0.04, 0.4, 4, 8 mg L^−1^	5-day seed germination test	Reduction of seed germination and root elongation	[58]
*Lactuca sativa*	Core–shell NPs Cu/CuO 13/9 nm	10, 20 mg L^−1^	15-days treatment of hydroponically grown lettuce	Reduction of water content, root length, and dry biomass of the plant, alteration of the nutritional quality of lettuce	[71]
*Raphanus sativus, Lolium perenne, Lolium rigidum*	CuO NPs 6 nm, CuO bulk particles 200 nm	10, 100, 500, 1000 mg L^−1^	The seeds were allowed to germinate for 6 days	Oxidative damage to plant DNA, inhibition of seedling growth (root and shoot growth)	[105]
*Elsholtzia splendens*	CuO NPs 34–52 nm, CuO bulk particles ˃1000 nm	100, 200, 500, 1000, 2000 mg L^−1^	Cu—tolerant plant, the seeds were allowed to germinate for 5 days, hydroponic experiments	No effect on seed germination, reduction of root length, accumulation of CuO NPs in root and leaf cells	[113]
*Zea mays, Oryza sativa*	CuO NPs 40–80 nm	500, 1000, 2000 mg L^−1^	Seed germination was investigated	CuO NPs inhibited root elongation at 2000 mg L^−1^ (95.73% for maize and 97.28% for rice) of two crop plants and reduced shoot length of maize by 30.98%	[91]

### Phytotoxicity of nanoparticles based on iron oxides

Most of the available studies on the phytotoxicity of iron nanomaterials have focused mainly on their advantages while relatively few have examined the mechanisms of phytotoxicity, uptake, translocation, and bioaccumulation [73]. Martinez-Fernandez et al. [106] investigated if water uptake by the roots could be affected by the adsorption of γ-Fe_2_O_3_ nanoparticles (50, 100 mg L^−1^) on the root surface of *Helianthus annuus*. The main effect was related to the reduction of the root hydraulic conductivity (Lo) and the nutrient uptake. The concentrations of the macronutrients Ca, K, Mg, and S in the shoot were reduced relative to the control plants, which resulted in lower contents of chlorophyll pigments. In the next study, the same group of authors [73] investigated the effects of nano zerovalent Fe (nZVI) and maghemite NPs (γ-Fe_2_O_3_) on the nutritional status of *S. lycopersicum*, through distinct effects on root functionality. A hydroponic experiment together with an incubation experiment helped to relate the reduction of the root water uptake with the potential blockage of root nutrient uptake by each nanomaterial. The treatment with 100 mg L^−1^ of γ-Fe_2_O_3_ inhibited 40% of the root hydraulic conductivity (Lo) of tomato plants (*S. lycopersicum* L.), which could explain the reduction in the Mo and Zn concentrations in their shoots. On the other hand, compared to γ-Fe_2_O_3_, nZVI seems to be less harmful since no effects on Lo were detected for the exposed roots, or regarding the shoot nutrient composition.

Liu et al. [83] investigated the effects of laboratory-prepared FeOx NPs (probably γ-Fe_2_O_3_) on the germination of lettuce (*L. sativa*) seeds in a water medium. NPs were not only less toxic than their ionic counterparts but also significantly stimulated the growth of root elongation by 12–26% in a concentration range (5–20 mg L^−1^). Conversely, at 50 mg L^−1^ root elongation was inhibited by 12%.

Gui et al. [107] performed a glasshouse study to quantify the uptake of γ-Fe_2_O_3_ NPs on transgenic and non-transgenic rice *O. sativa* plants. Nutrient concentrations, biomass, enzyme activity, and the concentration of two phytohormones, abscisic acid (ABA) and indole-3-acetic acid (IAA), and malondialdehyde (MDA) was measured. Root phytohormone inhibition was positively correlated with γ-Fe_2_O_3_ NP concentrations, indicating that Fe_2_O_3_ had a significant influence on the production of these hormones. The activities of antioxidant enzymes were significantly higher as a factor of low γ-Fe_2_O_3_ NP treatment concentration and significantly lower at high NPs concentrations, but only among transgenic plants. An overview of phytotoxicity of nanoparticles based on iron oxides is shown in Table 7.

**Table 7:** Phytotoxicity of nanoparticles based on iron oxides

Plant	Type of nanoparticle, particle size (nm)	Particle concentration	Comment	Observed effect	References
*Helianthus annuus*	γ-Fe_2_O_3_ 20–100 nm	50, 100 mg L^−1^	Effect on the root functionality was investigated	The treatment with 50 mg L^−1^ FeNPs significantly reduced the root hydraulic conductivity (*Lo*) by up to 26% at 100 mg L^−1^ FeNPs, but it had no effect on plant biomass production, on shoot or root elongation, and it did not induce oxidative stress in the plant	[106]
*Solanum lycopersicum*	nZVI < 50 nm γ-Fe_2_O_3_ 20–100 nm	50, 100 mg L^−1^	Effect on the root functionality was investigated	The treatment with 100 mg L^−1^ of Fe_2_O_3_ NPs inhibited 40% of the root hydraulic conductivity (*Lo*), with nZVI no effect on *Lo* was observed	[73]
*Lactuca sativa*	FeOx NPs <50 nm	1, 5, 10, 20, 50 mg L^−1^	A 5-day seed germination test was used to test how different FeOx NPs affected the plant growth in comparison with their respective ionic or solid counterparts	FeOx NPs significantly enhanced root elongation of lettuce seedlings by 12%–26%, indicating that FeOx NPs could be used as an Fe fertilizer as well at low application rates (5–20 mg L^−1^). At a concentration of 50 mg L^−1^, FeOx NPs decreased root elongation of lettuce seedlings by 20%	[83]
*Oryza sativa*	γ-Fe_2_O_3_ 7–13 nm	2, 20, 200 mg L^−1^	A 7-day seed germination test was used	Root phytohormone inhibition abscisic acid (ABA) and indole-3-acetic acid (IAA) was positively correlated with Fe_2_O_3_ NPs concentrations, indicating that Fe_2_O_3_ had a significant influence on the production of these hormones	[107]

## Conclusions

Phytotoxicity of any nanoparticle is largely influenced by its shape, size, chemical composition, and coating material composition. Sometimes, the phytotoxicity of nanoparticles may be as a result of the toxicity of substances, which were used for its preparation. Further, phytotoxicity may depend on the environment and on the physical and chemical nature of the plant species. The nanoparticles may have potentiating or inhibitory effects on plant growth in different developmental stages. Some nanoparticles are taken up by plant roots and transported to the aboveground parts of the plant through the vascular system, depending on the composition, shape, size of nanoparticle, and anatomy of the plant. Some nanoparticles remain adhered to the plant roots. In the discussed studies, sometimes nanoparticles have not been properly characterized and/or their composition vs. their shapes have not been considered, which is one the biggest obstacles need to be overcome for further planning of nanoparticles-plant research [108, 109]. Moreover, we have mentioned some papers and studies, where some metal based particles were both beneficial and toxic, but the right reason for these misleading findings lies in very high doses used together with a number of artifacts and misinterpretations especially regarding description of nanoparticles uptake. Despite the fact that a lot of knowledge has been acquired through many previous studies, many questions still remain unanswered such as the fate and behavior of nanoparticles in plant systems, or the role of surface area or activity of nanoparticles on phytotoxicity, and the role of plant cell walls in the internalization of nanoparticles.

In a study of phytotoxicity nanoparticles, the most urgent need is to build a connection between the characteristics of nanoparticles (surface area, particle size, surface tension) and phytotoxicity. Equally important is the need to understand the role of plant species and composition of the nanoparticles phytotoxicity. Finally, most studies on phytotoxicity and uptake of nanoparticles plants were performed in a hydroponic setup. Hydroponic studies do not reflect the interaction of nanoparticles with soil and soil microorganisms.

Finally, it can be concluded that the nanoparticles prepared from essential heavy metals and their oxides have proven to be suitable for use in the agriculture. The least phytotoxic of these appear to be nanoparticles made of iron oxides and manganese oxides.

## Abbreviations

ABA: abscisic acid
APX: ascorbate peroxidase
CAT: catalase
DHA: dehydrogenase activity
GDSL: motif consensus amino acid sequence of Gly, Asp, Ser, and Leu around the active site Ser
GDSL motif lipase 5: serine esterase and lipase with GDSL sequence motif
GS-GOGAT pathway: glutamine synthetase-glutamate synthase pathway
IAA: indole-3-acetic acid
ICP-OES: inductively coupled plasma optical emission spectrometry
MDA: malondialdehyde
NPs: nanoparticles
NR-NiR pathway: nitrate reductase-nitrite reductase pathway
POD: peroxidase
ROS: reactive oxygen species
SKU5 similar 4: multi-copper oxidase type I family protein expressed in plant roots
SOD: superoxide dismutase
SPIONs: superparamagnetic iron oxide nanoparticles
